# Supplementary material for: Content-rich biological network constructed by mining PubMed abstracts
Source: BMC Bioinformatics. 2004 Oct 8;5:147. doi: 10.1186/1471-2105-5-147 (PMC528731; doi:10.1186/1471-2105-5-147)
Supplement: Additional File 2 — The original results of the above study (non-essential files are deleted to keep the file size under the limit set by BMC bioinformatics). [file 1471-2105-5-147-S2.bz2 › chilibotAdditionalFile2/dip30/613ID10558980E10996/html/UBE3A_UBE2L3.html]

 


 **UBE3A** and **UBE2L3** 
  
Found 61 abstracts in PubMed, retrieved 30.  
 

 What does Google say? 
 PDF only 
| .edu only 

---

**Interactive relationship** (e.g. stimulation, inhibition, etc)

**Neutral relationship**- **UBE3A**  encodes  **UBE3A**  E6 AP  [ **UBE3A** ] , a ubiquitin protein ligase  [ **UBE2L3** ] , and shows brain specific imprinting, with brain expression predominantly from the maternal allele.  Ref: 11895368 Neurobiol Dis, 2002
- The crystal structure of the E6AP  [ **UBE3A** ]  hect domain bound to the UbcH7  [ **UBE2L3** ]  ubiquitin conjugating enzyme E2 reveals the determinants of E2 E3 specificity and provides insights into the transfer of ubiquitin from the E2 to the E3.  Ref: 10558980 Science, 1999
- also called ubiquitin protein ligase  [ **UBE2L3** ]  E3A  [ **UBE3A** ]  or  **UBE3A** , which functions as an ubiquitin protein ligase  [ **UBE2L3** ] .  Ref: 12619117 Hum Mutat, 2003
- Human papillomavirus HPV E6 protein forms a ternary complex with the cell cycle regulator p53 and the E6 associated protein E6AP  [ **UBE3A** ]  known as an E3 ubiquitin protein ligase  [ **UBE2L3** ] , leading to the degradation of p53 via the ubiquitination pathway.  Ref: 10974372 Antiviral Res, 2000
- The  **UBE3A**  gene, which codes for the enzyme ubiquitin protein ligase  [ **UBE2L3** ]  involved in protein degradation and processing, has been found to be mutated in many but not all of patients with Angelman syndrome and can be considered a major Angelman candidate gene.  Ref: 10514831 Adv Neurol, 1999
- We have isolated a mouse cDNA clone containing the C2 domain of mouse Nedd4, a ubiquitin protein ligase  [ **UBE2L3** ]  E3 that also contains a hect homologous to the E6 AP  [ **UBE3A** ]  carboxyl terminus domain and three WW domains.  Ref: 10446181 J Biol Chem, 1999
- The carboxyl terminal half of Rsp5 contains a HECT homologous to E6 AP  [ **UBE3A** ]  carboxyl terminus domain that catalytically ligates ubiquitin to proteins and functionally classifies Rsp5 as an E3 ubiquitin protein ligase  [ **UBE2L3** ] .  Ref: 10781604 J Biol Chem, 2000
- Studies aimed at the identification of cellular targets for E6AP  [ **UBE3A** ] , an E3 ubiquitin protein ligase  [ **UBE2L3** ]  involved in ubquitin mediated degradation, led us to the identification of members of the Src family kinases as potential substrates for E6AP  [ **UBE3A** ] .  Ref: 10449731 Proc Natl Acad Sci U S A, 1999

**Non-interactive relationship** (e.g. studied together, co-existance, homology, etc.)

- The E6 protein of the high risk human papillomaviruses HPVs and the cellular ubiquitin protein ligase  [ **UBE2L3** ]  E6AP  [ **UBE3A** ]  form a complex which causes the ubiquitination and degradation of p53.  Ref: 10864652 J Virol, 2000
- The E6 oncoprotein of human papillomaviruses HPVs that are associated with cervical cancer utilizes the cellular ubiquitin protein ligase  [ **UBE2L3** ]  E6 AP  [ **UBE3A** ]  to target the tumor suppressor p53 for degradation.  Ref: 11158620 Proc Natl Acad Sci U S A, 2001
- Identification of members of the Src tyrosine kinase family as substrates of the E6AP  [ **UBE3A** ]  ubiquitin protein ligase  [ **UBE2L3** ]  implicates a role for the ubiquitin pathway in regulating the activities of individual members of this important family of signaling molecules.  Ref: 10449731 Proc Natl Acad Sci U S A, 1999
- Mutations in the Ubiquitin protein ligase  [ **UBE2L3** ]  3A gene  **UBE3A**  have been found in the latter group, but only preliminary figures are available on their frequencies.  Ref: 10737998 Hum Mutat, 2000
- ... Patient 4 had a mutation in the ubiquitin protein ligase  [ **UBE2L3** ]   **UBE3A**  saving the beta3 subunit gene.  Ref: 11198279 Ann Neurol, 2001
- In addition, we also established that oxi 1, an oxidative stress responsive gene we previously cloned, encodes a family of proteins related to human E6 AP  [ **UBE3A** ]  ubiquitin protein ligase  [ **UBE2L3** ] .  Ref: 10408176 J Radiat Res Tokyo , 1999
- One candidate is the ubiquitin protein ligase  [ **UBE2L3** ]  gene  **UBE3A** , which is maternally expressed only and localized to the 15q region.  Ref: 11489053 J Intellect Disabil Res, 2001
- 4 a mutation in the E3 ubiquitin protein ligase  [ **UBE2L3** ]  gene  **UBE3A** .  Ref: 11748306 J Med Genet, 2001
- Structure of an E6AP  [ **UBE3A** ]  UbcH7  [ **UBE2L3** ]  complex insights into ubiquitination by the E2 E3 enzyme cascade.  Ref: 10558980 Science, 1999
- We identified a number of positive interacting clones that encode ubiquitin protein ligase  [ **UBE2L3** ]  E3A  [ **UBE3A** ]   **UBE3A** .  Ref: 12207887 Biochem Biophys Res Commun, 2002
- Human scribble Vartul is targeted for ubiquitin mediated degradation by the high risk papillomavirus E6 proteins and the E6AP  [ **UBE3A** ]  ubiquitin protein ligase  [ **UBE2L3** ] .  Ref: 11027293 Mol Cell Biol, 2000
- It results from a maternal deletion of human chromosome 15q11 13 containing two candidate genes E6 P ubiquitin protein ligase  [ **UBE2L3** ]   **UBE3A**  and GABA A receptor beta3 subunit GABRB3, the latter of which has been also linked to autism.  Ref: 12672542 Neurosci Lett, 2003
- Maternal truncation mutations in  **UBE3A** , which encodes for E6 AP  [ **UBE3A** ]  ubiquitin protein ligase  [ **UBE2L3** ] , have been shown to cause Angelman syndrome, which can also result from the absence of maternal chromosomal material from this region.  Ref: 10089011 Mol Psychiatry, 1999
- VCY2 protein interacts with the HECT domain of ubiquitin protein ligase  [ **UBE2L3** ]  E3A  [ **UBE3A** ] .  Ref: 12207887 Biochem Biophys Res Commun, 2002
- Members of the hect domain protein family are characterized by sequence similarity of their C terminal regions to the C terminus of E6 AP  [ **UBE3A** ] , an E3 ubiquitin protein ligase  [ **UBE2L3** ] .  Ref: 10066826 J Biol Chem, 1999
- The high risk human papillomavirus HPV E6 proteins stimulate the ubiquitination and degradation of p53, dependent on the E6AP  [ **UBE3A** ]  ubiquitin protein ligase  [ **UBE2L3** ] .  Ref: 11027293 Mol Cell Biol, 2000
- The human papilloma virus E6 associated protein E6AP  [ **UBE3A** ]  functions as a ubiquitin protein ligase  [ **UBE2L3** ]  E3 in the E6 mediated ubiquitination of p53.  Ref: 10373495 J Biol Chem, 1999
- On the basis of discoveries made in molecular biology, patients can be classified as belonging to five types deletion, paternal uniparental disomy UPD, imprinting defects, mutation of the  **UBE3A**  ubiquitin protein ligase  [ **UBE2L3** ]  gene and unidentified mechanism 15% 20% of patients.  Ref: 12373673 Rev Neurol, 2002
- Typical NEDD4 orthologues that contain a C2 Ca 2 lipid binding and a HECT Homologous to the E6 AP  [ **UBE3A** ]  Carboxyl Terminus ubiquitin protein ligase  [ **UBE2L3** ]  domain, and multiple WW domains have been shown to regulate the epithelial sodium channel ENaC.  Ref: 11840194 Eur J Hum Genet, 2001
- Human papillomavirus type 16 E6 induces self ubiquitination of the E6AP  [ **UBE3A** ]  ubiquitin protein ligase  [ **UBE2L3** ] .  Ref: 10864652 J Virol, 2000
- The E6AP  [ **UBE3A** ]  ubiquitin protein ligase  [ **UBE2L3** ]  E3 mediates the human papillomavirus induced degradation of the p53 tumor suppressor in cervical cancer and is mutated in Angelman syndrome, a neurological disorder.  Ref: 10558980 Science, 1999
- A candidate AS gene, ubiquitin protein ligase  [ **UBE2L3** ]  3A  **UBE3A**  E6 AP  [ **UBE3A** ] , has been identified, and mutations of this gene have been detected in several cases of AS.  Ref: 10732796 Neurogenetics, 1998
- ... E6AP  [ **UBE3A** ]  accepted ubiquitins from Ubc4, UbcH5B, UbcH5C and UbcH7  [ **UBE2L3** ] .  Ref: 9990509 Genes Cells, 1998
